# New Insights into the Role of SGLT-2 Inhibitors in the Prevention of Dementia

**DOI:** 10.3390/neurolint16060124

**Published:** 2024-12-05

**Authors:** Cheng-Hsien Hung, Li-Yu Lu

**Affiliations:** 1 Department of Pharmacy, Chang Bing Show Chwan Memorial Hospital, Changhua 50544, Taiwan; 2 Institute of Medicine, Chung Shan Medical University, Taichung 40201, Taiwan; 3 School of Medicine, Chung Shan Medical University, Taichung 40201, Taiwan; liyu061437@gmail.com

**Keywords:** SGLT-2 inhibitors, gliflozins, dementia, diabetes mellitus, Alzheimer’s disease

## Abstract

Diabetes mellitus (DM) is a chronic disease associated with numerous complications, including cardiovascular diseases, nephropathy, and neuropathy. Sodium–glucose cotransporter 2 (SGLT-2) inhibitors, a class of novel antidiabetic agents, have demonstrated promising therapeutic effects beyond glycemic control, with potential benefits extending to the cardiovascular and renal systems. Recently, research has increasingly focused on exploring the potential role of SGLT-2 inhibitors in preventing dementia. The aim of this review is to summarize the current research suggesting that SGLT-2 inhibitors, such as empagliflozin and dapagliflozin, may have neuroprotective effects that reduce dementia risk and improve cognitive function in type 2 diabetes patients. These benefits are likely due to better glycemic control, reduced oxidative stress, and less advanced glycation end-product (AGE) formation, all linked to neurodegeneration. Despite these promising findings, existing studies are limited by small sample sizes and short follow-up durations, which may not adequately capture long-term outcomes. To establish more robust evidence, larger-scale, long-term randomized controlled trials (RCTs) involving diverse populations are needed. These studies should involve diverse populations and focus on understanding the mechanisms behind the neuroprotective effects. Addressing these limitations will provide clearer guidelines for using SGLT-2 inhibitors in dementia prevention and management. This will help improve therapeutic strategies for cognitive health in diabetic patients.

## 1. Introduction

### 1.1. Global Prevalence of Diabetes Mellitus and Its Complications

Diabetes mellitus (DM) has become one of the most prevalent chronic diseases worldwide [1], affecting millions of individuals. According to the International Diabetes Federation (IDF), the global prevalence of diabetes in 2019 was estimated at 463 million [2], with projections indicating that this number will rise to 700 million by 2045. This alarming increase is attributed to various factors, including aging populations [3], urbanization [4], unhealthy diets [5], and sedentary lifestyles [6].

The complications of diabetes are numerous and severe, impacting multiple organ systems. Cardiovascular diseases (CVDs) are the leading cause of morbidity and mortality among diabetic patients [7], accounting for nearly 70% of all diabetes-related deaths. Diabetic nephropathy, characterized by progressive kidney damage, is the leading cause of end-stage renal disease (ESRD) worldwide [8]. Diabetic neuropathy, which affects the peripheral nerves, leads to significant morbidity [9], including pain, loss of sensation, and increased risk of foot ulcers and amputations [10]. Additionally, diabetic retinopathy, a leading cause of blindness, affects nearly one-third of diabetic patients [10].

### 1.2. Discovery and Development of SGLT-2 Inhibitors

The discovery and development of SGLT-2 inhibitors marked a significant advancement in diabetes treatment [11]. The sodium–glucose cotransporter 2 (SGLT-2) is a protein located in the proximal renal tubules [12], responsible for reabsorbing approximately 90% of the filtered glucose back into the bloodstream. Inhibiting this transporter reduces glucose reabsorption, resulting in increased urinary glucose excretion and lower blood glucose levels [13].

The development of SGLT-2 inhibitors began in the early 2000s, with the identification of the transporter’s role in glucose homeostasis [14]. The first SGLT-2 inhibitor, Canagliflozin, was approved by the U.S. Food and Drug Administration (FDA) in 2013 [15]. Since then, several other SGLT-2 inhibitors, including empagliflozin, canagliflozin, and ertugliflozin, have been developed and approved for clinical use.

### 1.3. Protective Effects of SGLT-2 Inhibitors on the Heart and Kidneys

Beyond their role in glucose regulation, SGLT-2 inhibitors have demonstrated remarkable protective effects on both the heart and kidneys [16,17], making them valuable in managing complications associated with diabetes. These inhibitors have been shown to reduce the risk of cardiovascular events in patients with type 2 diabetes. Clinical trials such as the EMPA-REG OUTCOME and CANVAS studies have highlighted the cardioprotective benefits of SGLT-2 inhibitors [18], including reductions in major adverse cardiovascular events (MACE), heart failure hospitalizations, and overall cardiovascular mortality [19]. The mechanism behind this cardioprotection is multifactorial, involving improved glycemic control, reduction in blood pressure, diuresis, and favorable effects on myocardial energy metabolism [20].

In addition to cardiovascular benefits, SGLT-2 inhibitors have also shown promise in preserving kidney function. Studies like CREDENCE and DAPA-CKD have demonstrated that these agents significantly reduce the risk of kidney disease progression, particularly in patients with diabetic nephropathy [21]. By lowering intraglomerular pressure, reducing albuminuria, and promoting natriuresis, SGLT-2 inhibitors provide renoprotection, which is critical given that diabetic nephropathy is a leading cause of end-stage renal disease [22]. These dual benefits for both the heart and kidneys suggest that SGLT-2 inhibitors are not only effective for glycemic control but also for mitigating the complications of diabetes, particularly in the context of cardiovascular and renal health.

### 1.4. Pathophysiology of Dementia and Its Link to Diabetes

Dementia is a complex neurodegenerative disorder characterized by a progressive decline in cognitive function, affecting memory, thinking, behavior, and the ability to perform everyday activities [23]. The most common form of dementia is Alzheimer’s disease (AD), which accounts for 60–80% of cases [24]. Other forms include vascular dementia, dementia with Lewy bodies, and frontotemporal dementia.

The pathophysiology of dementia involves multiple mechanisms, including the accumulation of amyloid-beta (Aβ) plaques [25], hyperphosphorylation of tau proteins leading to neurofibrillary tangles, synaptic dysfunction, and neuronal loss. Inflammatory processes [26], oxidative stress [27], mitochondrial dysfunction, and impaired glucose metabolism also play significant roles in the progression of dementia [28,29,30]. Recent research also identified other factors influencing the progression of dementia, such as disruptions in the kynurenine pathway [31].

**Figure 1:**
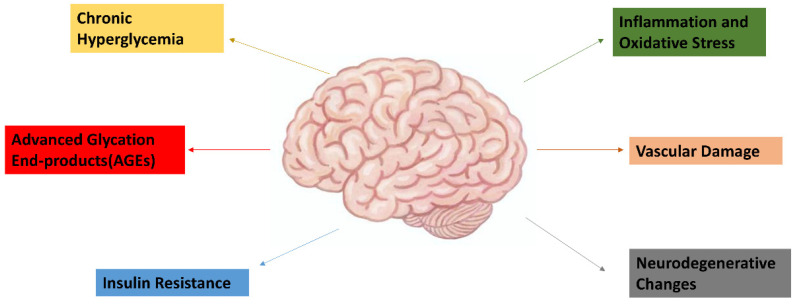
Pathophysiological mechanisms linking type 2 diabetes and dementia. Chronic hyperglycemia triggers the formation of advanced glycation end-products (AGEs), insulin resistance, and vascular damage, all of which play a role in cognitive decline. Additionally, inflammation and oxidative stress further exacerbate vascular and neurodegenerative changes, accelerating the progression toward dementia.

Epidemiological studies have established a strong link between diabetes and an increased risk of dementia [32]. Previous systematic reviews had also found a significant association between diabetes and the risk of cognitive impairment and dementia, with diabetic patients having a 1.25–1.91-fold increased risk [33]. Furthermore, prediabetes was associated with an elevated risk of developing dementia. Various diabetes-related biochemical markers, including fasting plasma glucose, 2 h post-load glucose, glycated hemoglobin (HbA1c), and irregular fasting plasma insulin levels, were also linked to a heightened dementia risk [33]. Several mechanisms contribute to this association, including chronic hyperglycemia, insulin resistance, advanced glycation end-products (AGEs), and vascular damage [34]. Hyperglycemia and insulin resistance impair insulin signaling in the brain, leading to reduced neuronal glucose uptake and energy deficits [35]. AGEs and oxidative stress promote inflammation and neuronal damage [36]. Vascular complications, such as microvascular and macrovascular disease, contribute to cerebral ischemia and hypoxia, exacerbating cognitive decline [37] (Figure 1).

### 1.5. Existing Treatments for Dementia and Their Limitations

Current treatments for dementia are primarily symptomatic, aiming to alleviate cognitive and behavioral symptoms rather than modifying the disease course. The main classes of medications include cholinesterase inhibitors [38] (donepezil, rivastigmine, galantamine) and the NMDA receptor antagonist memantine. Cholinesterase inhibitors work by increasing the levels of acetylcholine, a neurotransmitter involved in memory and learning, while memantine modulates glutamatergic activity to prevent excitotoxicity [39]. There was also non-invasive brain stimulation in the treatment of Alzheimer’s disease that was under research [40,41,42].

Despite their benefits, these treatments have limitations. They provide modest symptomatic relief and do not alter the underlying disease pathology. The effects are often temporary, with diminishing efficacy over time [43]. Additionally, these medications are associated with side effects, such as gastrointestinal disturbances, bradycardia, and dizziness, which can limit their use [44].

Despite significant advances in understanding dementia’s pathophysiology, there is still no definitive treatment for preventing its onset. Researchers have explored various lifestyles and pharmacological interventions aimed at reducing the risk of dementia, with mixed results. Lifestyle modifications, including regular physical exercise, maintaining a healthy diet, cognitive training, and managing cardiovascular risk factors (such as hypertension and diabetes), have shown potential in observational studies for delaying or reducing the risk of dementia development [45,46]. However, these strategies are not universally effective, and their specific impact on dementia prevention is still debated. While these interventions appear promising, randomized controlled trials (RCTs) have not yet conclusively demonstrated that any particular lifestyle or pharmacological intervention can reliably prevent dementia. As a result, no standardized prevention strategy has been established. This highlights the pressing need for more rigorous research into preventive measures, with a focus on understanding the modifiable risk factors that contribute to the development of dementia [47].

Given these limitations, there is an urgent need for novel strategies that target the underlying mechanisms of dementia. SGLT-2 inhibitors have emerged as potential candidates due to their pleiotropic effects, which extend beyond glucose control to include neuroprotective and anti-inflammatory properties [48,49].

## 2. The Neuroprotective Potential of SGLT-2 Inhibitors

### 2.1. Early Animal Studies on the Neuroprotective Effects of SGLT-2 Inhibitors

The potential neuroprotective effects of SGLT-2 inhibitors in dementia were first explored in early preclinical studies using animal models. The detailed results are summarized in Table 1. These studies provided promising evidence that SGLT-2 inhibitors could ameliorate cognitive decline and protect against neurodegeneration. In rodent models of Alzheimer’s disease, treatment with SGLT-2 inhibitors such as empagliflozin and dapagliflozin showed significant improvements in memory and learning abilities [50]. These benefits were thought to be linked to the drugs’ ability to reduce hyperglycemia and insulin resistance, key factors implicated in both diabetes and neurodegenerative processes.

**Table 1:** Evidence from animal models: SGLT2 inhibitors and neuroprotective effects.

Study	SGLT-2 Type	Main Mechanisms	Key Findings
Yaribeygi et al. (2024) [50]	Empagliflozin, Dapagliflozin	- Reduction in hyperglycemia and insulin resistance - Improvement of glucose metabolism	- SGLT-2 inhibitors may slow cognitive decline and protect against neurodegeneration
Khamieset et al. (2024) [51]	Empagliflozin	- Reduction in amyloid-beta (Aβ) plaque accumulation - Improved synaptic function - Enhanced neuronal survival	- Empagliflozin improved memory and learning abilities, reducing Alzheimer’s disease pathology
Chmiel et al. (2023) [52]	Dapagliflozin	- Reduction in oxidative stress - Decrease in pro-inflammatory cytokines (e.g., IL-1β, TNF-α) - Reduced neuroinflammation	- Dapagliflozin lowered neuroinflammation and related pro-inflammatory responses

Abbreviation: SGLT-2: Sodium–glucose cotransporter 2; IL: Interleukin; TNF: Tumor necrosis factor.

Furthermore, these studies revealed that SGLT-2 inhibitors could attenuate the accumulation of amyloid-beta (Aβ) plaques, a hallmark of Alzheimer’s disease [51]. By improving glucose metabolism and reducing oxidative stress, SGLT-2 inhibitors were shown to enhance neuronal survival and prevent synaptic dysfunction. Other mechanisms observed in animal studies included reductions in neuroinflammation and decreased levels of pro-inflammatory cytokines, which are thought to play a critical role in the pathogenesis of dementia [52]. These early findings suggest that SGLT-2 inhibitors may not only address metabolic dysfunction but also target underlying neurodegenerative processes, positioning them as potential candidates for dementia prevention and treatment.

### 2.2. The Neuroprotective Effects of SGLT-2 Inhibitors in Human Studies

A comprehensive review of studies reveals a consistent trend towards reduced dementia risk with SGLT-2 inhibitor use. For instance, a study by Wu et al. (2023) found that patients treated with dapagliflozin had a significantly lower incidence of Alzheimer’s disease compared to those on standard antidiabetic therapy [53]. Another study by Bujalance et al. (2020) reported that empagliflozin users exhibited a reduced risk of vascular dementia, highlighting the neuroprotective potential of SGLT-2 inhibitors across different dementia subtypes [54].

Several meta-analyses have pooled data from multiple studies to evaluate the impact of SGLT-2 inhibitors on dementia risk. A meta-analysis by Youn et al. (2024) included 12 studies with a total of 45,089 participants and reported a pooled hazard ratio of 0.68 (95% CI: 0.50–0.92) for dementia incidence among SGLT-2 inhibitor users [55]. Similarly, a meta-analysis by Tang et al. (2023) found that SGLT-2 inhibitors were associated with a significant reduction in the risk of both Alzheimer’s disease and vascular dementia [56].

Real-world evidence from observational studies supports the findings of clinical trials and meta-analyses. A large cohort study by Siao et al. (2022) involving 976,972 diabetic patients found that those treated with SGLT-2 inhibitors had a lower risk of developing dementia compared to those on other glucose-lowering medications. These findings highlight the potential of SGLT-2 inhibitors to provide neuroprotection in a real-world clinical setting [57].

## 3. Mechanisms of Action

### 3.1. Molecular Mechanisms of SGLT-2 Inhibitors

**Figure 2:**
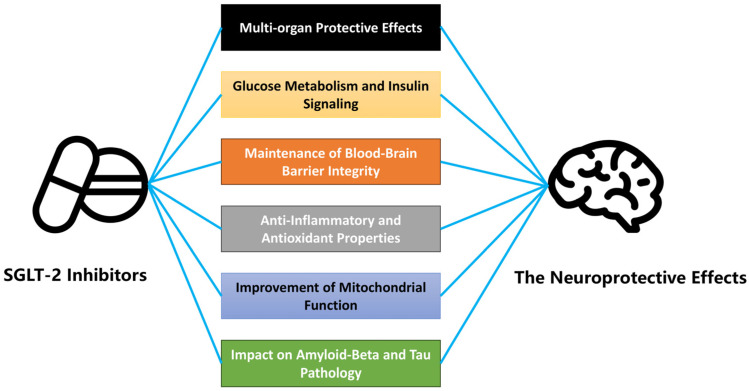
Mechanisms underlying the neuroprotective effects of SGLT-2 inhibitors. This figure illustrates the proposed mechanisms by which SGLT-2 inhibitors may exert neuroprotective effects. These mechanisms include multi-organ protective effects that indirectly support brain function and enhanced glucose metabolism and insulin signaling, which improve neuronal health. SGLT-2 inhibitors also help maintain the integrity of the blood–brain barrier, preventing neurotoxic insults, and exhibit anti-inflammatory and antioxidant properties, which reduce neuroinflammation and oxidative stress. Furthermore, they promote mitochondrial function by improving energy production and reducing mitochondrial dysfunction. Lastly, SGLT-2 inhibitors may attenuate pathological hallmarks of Alzheimer’s disease, such as amyloid-beta and tau pathology. The arrows depict the connections between SGLT-2 inhibitors and their specific roles in achieving these neuroprotective effects.

Sodium–glucose cotransporter 2 (SGLT-2) inhibitors primarily lower blood glucose by inhibiting the reabsorption of glucose in the proximal renal tubules of the kidneys [58]. Specifically, these drugs block the SGLT-2 transporter, which is responsible for reabsorbing approximately 90% of filtered glucose back into the bloodstream. By inhibiting this transporter, SGLT-2 inhibitors induce glycosuria, thereby reducing plasma glucose levels [59]. However, their effects are not confined to glucose lowering. Emerging research suggests that SGLT-2 inhibitors exert neuroprotective effects through multiple mechanisms, many of which are independent of glycemic control. These include improvements in glucose metabolism, reductions in oxidative stress, preservation of blood–brain barrier integrity, modulation of inflammatory responses, and beneficial impacts on mitochondrial function and amyloid-beta and tau pathology, all of which contribute to their neuroprotective potential in patients with type 2 diabetes mellitus (T2DM) and cognitive decline (Figure 2).

### 3.2. Effects on Glucose Metabolism and Insulin Signaling

Hyperglycemia and insulin resistance are strongly associated with cognitive decline and dementia in individuals with diabetes. Chronic hyperglycemia impairs glucose utilization in the brain, leading to energy deficits and neuronal dysfunction. SGLT-2 inhibitors improve glycemic control by reducing blood glucose levels and enhancing insulin sensitivity, thereby mitigating the detrimental effects of hyperglycemia on neuronal cells. This improvement in glucose metabolism has a downstream effect on reducing the formation of advanced glycation end-products (AGEs), which are proteins or lipids that become glycated as a result of prolonged exposure to high sugar levels. AGEs accumulate in various tissues, including the brain, where they contribute to oxidative stress and inflammation—key factors in neurodegeneration [60]. SGLT-2 inhibitors not only reduce AGEs but also lower oxidative stress by decreasing the production of reactive oxygen species (ROS) in neurons. This improvement in glucose homeostasis may help protect against the metabolic and inflammatory stress that contributes to cognitive decline in T2DM patients.

### 3.3. Blood–Brain Barrier Integrity and Neuroprotection

The blood–brain barrier (BBB) plays a vital role in maintaining brain homeostasis by selectively allowing essential nutrients to pass into the brain while preventing the entry of harmful substances. Hyperglycemia, oxidative stress, and chronic inflammation can compromise BBB integrity, leading to increased permeability and the infiltration of neurotoxic molecules, such as pro-inflammatory cytokines and ROS, into brain parenchyma. This disruption of BBB function contributes to neuroinflammation, neuronal damage, and cognitive decline [61]. Studies have demonstrated that SGLT-2 inhibitors may protect BBB integrity by reducing glucose-related oxidative stress and inflammation. For example, dapagliflozin and empagliflozin have been shown to preserve tight junction proteins, which are crucial for maintaining the BBB’s selective permeability [62]. By safeguarding the BBB, SGLT-2 inhibitors may prevent the influx of harmful substances into the brain, thus reducing the risk of neurodegeneration and cognitive decline in patients with diabetes.

### 3.4. Anti-Inflammatory and Antioxidant Properties

Inflammation and oxidative stress are critical drivers of neurodegenerative diseases, including Alzheimer’s disease. Chronic hyperglycemia and insulin resistance trigger systemic inflammation, leading to the release of pro-inflammatory cytokines, such as interleukin-6 (IL-6) and tumor necrosis factor-alpha (TNF-α) [63]. These cytokines exacerbate neuronal damage by promoting neuroinflammation and oxidative stress within the brain. SGLT-2 inhibitors have demonstrated anti-inflammatory effects in both clinical and preclinical studies. For instance, empagliflozin has been shown to reduce serum levels of IL-6 and TNF-α in diabetic mice, thereby decreasing neuroinflammation and oxidative stress. Additionally, these inhibitors boost the expression of antioxidant enzymes, such as superoxide dismutase (SOD) and catalase, which neutralize ROS and protect neurons from oxidative damage [64]. This dual anti-inflammatory and antioxidant action contributes to the neuroprotective effects of SGLT-2 inhibitors, potentially slowing the progression of cognitive decline and neurodegenerative processes in patients with diabetes.

### 3.5. Mitochondrial Function and Neuroprotection

Mitochondrial dysfunction is a hallmark of many neurodegenerative diseases, including Alzheimer’s and Parkinson’s diseases. Neurons are particularly vulnerable to mitochondrial defects due to their high energy demands. Impaired mitochondrial function results in reduced ATP production and increased production of ROS, which contributes to oxidative stress and neuronal death [65]. SGLT-2 inhibitors have been found to improve mitochondrial function in both peripheral tissues and the brain. Empagliflozin, in particular, has been shown to enhance mitochondrial biogenesis, increase ATP production, and reduce ROS in the brains of diabetic mice, thereby improving neuronal survival [66]. By improving mitochondrial efficiency and reducing oxidative stress, SGLT-2 inhibitors may protect against the energy deficits and oxidative damage that are key contributors to cognitive impairment in diabetic patients.

### 3.6. Impact on Amyloid-Beta and Tau Pathology

The accumulation of amyloid-beta (Aβ) plaques and hyperphosphorylated tau proteins is a hallmark of Alzheimer’s disease pathology. These neurotoxic aggregates impair synaptic function, disrupt neuronal communication, and lead to neuronal death, all of which contribute to cognitive decline. Preclinical studies suggest that SGLT-2 inhibitors may directly influence amyloid and tau pathology. For example, dapagliflozin has been shown to reduce Aβ deposition and tau hyperphosphorylation in the brains of diabetic mice, potentially through its effects on glucose metabolism, insulin signaling, and neuroinflammation [67]. By reducing the production and aggregation of these toxic proteins, SGLT-2 inhibitors may help preserve synaptic function and prevent cognitive decline. Furthermore, the reduction in oxidative stress and inflammation induced by SGLT-2 inhibitors may further attenuate the pathological processes leading to amyloid and tau aggregation. This multifaceted impact on Alzheimer’s disease pathology positions SGLT-2 inhibitors as promising candidates for the prevention or slowing of neurodegenerative processes in patients with diabetes.

## 4. Clinical Evidence

Several population-based cohort studies have explored the association between sodium–glucose cotransporter 2 (SGLT2) inhibitors and the risk of dementia in patients with type 2 diabetes mellitus (T2DM), revealing their potential neuroprotective effects. The detailed findings are presented in Table 2.

**Table 2:** Key findings from studies on SGLT2 inhibitors and dementia risk.

Study	Population	Sample Size	Intervention	Outcome	Results
Wu et al. (2023) [53]	Patients aged ≥ 66 years with diabetes in Canada	106,903	SGLT2 inhibitors vs. DPP-4 inhibitors	Time to incident dementia	SGLT2 inhibitors associated with a lower risk of dementia compared to DPP-4
Siao et al. (2022) [57]	Patients with type 2 diabetes in Taiwan	206,494	SGLT2 inhibitors vs. non-SGLT2 inhibitors	Incident dementia	SGLT2 inhibitor group had a lower risk of incident dementia
Chen et al. (2024) [68]	Diabetic patients with AF in Taiwan	2430	SGLT2 inhibitors vs. non-SGLT2 inhibitors	Incident dementia	SGLT2 inhibitors associated with reduced risks of incident dementia
Perna et al. (2018) [69]	Elderly (>65 years of age) men and women with T2DM in Italy	39	SGLT2 inhibitors vs. incretins	Cognitive status change	Cognitive status did not change significantly during the 12 months of treatment in either of the groups
Mui et al. (2021) [70]	Type 2 diabetes mellitus patients in Hong Kong	39,828	SGLT2 inhibitors vs. DPP-4 inhibitors	New-onset dementia	The use of SGLT2 inhibitors is associated with a significantly lower risk of dementia
Shin et al. (2024) [71]	Type 2 diabetes mellitus patients aged 40–69 in Korea	221,770	SGLT2 inhibitors vs. DPP-4 inhibitors	New-onset dementia	The use of SGLT2 inhibitors is associated with a significantly lower risk of dementia hazard ratios of 0.65 (95% CI 0.58–0.73)

Abbreviation: SGLT2: Sodium–glucose cotransporter 2; DPP-4: Dipeptidyl peptidase-4; T2DM: type 2 diabetes mellitus.

One retrospective cohort study from Ontario focused on individuals aged 66 and older and compared new users of SGLT2 inhibitors with users of dipeptidyl peptidase-4 (DPP-4) inhibitors. The study found a 20% reduction in dementia risk among SGLT2 inhibitor users (adjusted hazard ratio [aHR]: 0.80, 95% CI: 0.71–0.89), with dapagliflozin showing the most significant neuroprotective effect [53].

A nationwide cohort study conducted in Taiwan further supported these findings. This study, which included 103,247 new users of SGLT2 inhibitors, demonstrated a significant reduction in dementia risk (aHR: 0.89, 95% CI: 0.82–0.96). This study suggests that SGLT2 inhibitors may play a critical role in reducing cognitive decline in T2DM patients, warranting further investigation through clinical trials to confirm these observations [57].

In diabetic patients with atrial fibrillation (AF), SGLT2 inhibitors have also been shown to reduce both the risk of dementia and cardiovascular events. A nationwide cohort from Taiwan involving 810 SGLT2 inhibitor users revealed a 29% lower risk of developing dementia (HR: 0.71, 95% CI: 0.51–0.98), particularly vascular dementia (HR: 0.44, 95% CI: 0.24–0.82), compared to non-users. Additionally, SGLT2 inhibitor users exhibited lower rates of AF-related hospitalizations, stroke, and all-cause mortality, suggesting protective benefits for both cognitive and cardiovascular health [68].

A 12-month randomized controlled trial compared SGLT2 inhibitors with incretin-based therapies in elderly patients with T2DM in Italy. Although no significant differences were observed in cognitive performance between the two groups, those receiving SGLT2 inhibitors showed notable improvements in metabolic outcomes, such as reductions in body weight and BMI, as well as increases in HDL cholesterol levels. These findings indicate that while both treatments preserve cognitive function, SGLT2 inhibitors may offer additional metabolic benefits [69].

In another cohort study including type 2 diabetes mellitus patients in Hong Kong, SGLT2 inhibitors were found to significantly reduce the risk of new-onset dementia compared to DPP-4 inhibitors. SGLT2 inhibitor users experienced a lower risk of developing dementia (HR: 0.41, 95% CI: 0.27–0.61). Furthermore, the use of SGLT2 inhibitors was associated with lower incidences of Alzheimer’s and Parkinson’s diseases, as well as reduced all-cause, cardiovascular, and cerebrovascular mortality, underscoring their potential neuroprotective benefits [70].

We found a study analyzing 724 young adults aged 18–35 to investigate the relationship between metabolic syndrome (MS) components and oxidative stress (OS) parameters, using principal component analysis (PCA) to identify the most significant contributors. Results revealed that individuals with MS exhibited significantly lower superoxide dismutase (SOD) activity (including MnSOD and CuZnSOD), higher total antioxidant capacity (TAC), and reduced thiol group concentrations compared to non-MS individuals. PCA identified three main components of MS: “Obesity and insulin resistance”, “Dyslipidemia”, and “Blood pressure”, with “Obesity and insulin resistance” showing the strongest correlation with OS parameters. This component was negatively associated with SOD activity, indicating a profound impact on reducing antioxidant defense. The “Dyslipidemia” component correlated most strongly with TAC, suggesting that lipid abnormalities influence overall antioxidant capacity, while the “Blood pressure” component had a weaker association with OS but may contribute to vascular oxidative damage. By quantifying the relative contributions of these components, the study highlights the dominant role of obesity and insulin resistance in driving OS in MS. These findings underscore the need for targeted interventions focusing on obesity and insulin resistance to mitigate OS-related damage and improve clinical outcomes for MS patients. This novel approach provides new insights into the pathophysiology of MS and potential therapeutic strategies [72].

Finally, a large-scale cohort study in Korea assessed the risk of dementia in adults aged 40–69 years with T2DM who were treated with SGLT2 inhibitors versus DPP-4 inhibitors. The study, which included 110,885 matched pairs, followed participants for an average of 670 days and found a 35% reduction in dementia risk among SGLT2 inhibitor users (HR: 0.65, 95% CI: 0.58–0.73). This protective effect was consistent across different types of dementia and subgroups, reinforcing the hypothesis that SGLT2 inhibitors may offer broad neuroprotective effects in middle-aged T2DM patients [71].

The long-term efficacy of sodium–glucose cotransporter 2 (SGLT-2) inhibitors in managing type 2 diabetes mellitus (T2DM) extends beyond glycemic control, with growing evidence supporting their role in reducing long-term cardiovascular and renal complications. Long-term studies, such as the EMPA-REG OUTCOME and CANVAS trials, have demonstrated sustained reductions in cardiovascular mortality, heart failure hospitalizations, and progression to renal disease with continued use of SGLT-2 inhibitors [19,73]. These trials followed patients for an average of 3.1–4.2 years, showing that the cardioprotective and renoprotective benefits were maintained over time, even in patients with high cardiovascular risk profiles. Importantly, these effects were independent of glycemic control, indicating that SGLT-2 inhibitors have pleiotropic benefits that extend beyond blood sugar regulation [74]. These findings suggest that long-term use of SGLT-2 inhibitors not only manages hyperglycemia but also mitigates the complications of diabetes, improving overall patient outcomes.

The long-term safety profile of SGLT-2 inhibitors has been extensively studied, with a generally favorable safety record, though certain risks have been noted. While SGLT-2 inhibitors are associated with an increased risk of genital infections due to glucosuria, these events are typically mild and manageable [75]. Studies have also highlighted a slightly elevated risk of diabetic ketoacidosis (DKA), particularly in patients with type 1 diabetes or those on insulin therapy, though the incidence remains low in T2DM patients [76]. Importantly, the risk of bone fractures, initially raised as a concern in the CANVAS trial, has not been consistently observed across all studies, with subsequent data indicating that fracture risk may not be significantly increased with SGLT-2 inhibitors [21]. Moreover, the long-term renal safety of these agents is well supported, as evidenced by the CREDENCE trial, which demonstrated a significant reduction in renal disease progression without major adverse effects on kidney function [21]. Overall, the benefits of long-term SGLT-2 inhibitor use appear to outweigh the risks, particularly when managed appropriately in clinical practice.

## 5. Discussion

### 5.1. Future Directions

SGLT-2 inhibitors, such as empagliflozin and dapagliflozin, are primarily used for their glucose-lowering effects by inhibiting renal sodium–glucose cotransport, increasing urinary glucose excretion, and reducing blood glucose levels [77]. Beyond glycemic control, these drugs show potential neuroprotective effects in preclinical and clinical studies [78].

Chronic hyperglycemia and insulin resistance in type 2 diabetes contribute to cognitive decline and dementia [79]. Hyperglycemia leads to the formation of advanced glycation end-products (AGEs) and oxidative stress, which promote neurodegeneration [80]. SGLT-2 inhibitors improve glycemic control, enhance insulin sensitivity, and reduce AGEs and oxidative stress, potentially mitigating cognitive decline.

Hyperglycemia and systemic inflammation in diabetes can compromise blood–brain barrier (BBB) integrity, allowing harmful substances to enter the brain and exacerbate neurodegeneration. SGLT-2 inhibitors help preserve BBB integrity by reducing inflammation and oxidative stress, offering a neuroprotective mechanism [81].

Mitochondrial dysfunction, a hallmark of neurodegenerative diseases, is improved by SGLT-2 inhibitors, which enhance mitochondrial biogenesis and reduce oxidative stress. For instance, empagliflozin has been shown to decrease reactive oxygen species production in neuronal cells [82,83]. Additionally, these drugs may reduce amyloid-beta production, aggregation, and tau hyperphosphorylation, critical steps in Alzheimer’s disease pathology [84].

Meta-analyses have consistently linked SGLT-2 inhibitor use with reduced dementia risk. For example, Youn et al. reported a pooled hazard ratio of 0.68 for dementia incidence, indicating significant protection [55]. These findings suggest that SGLT-2 inhibitors may improve glycemic control while reducing cognitive decline in diabetic patients.

### 5.2. Limitation

However, current evidence has limitations. Most studies have short follow-up periods, potentially missing long-term effects. The precise mechanisms of neuroprotection remain unclear, and long-term randomized controlled trials are needed to confirm these benefits.

Many existing studies vary in design, including differences in patient populations, dosage regimens, and outcome measures. For example, some studies focus exclusively on Alzheimer’s disease, while others include vascular dementia or mixed types. This heterogeneity makes it difficult to draw generalizable conclusions about the broad efficacy of SGLT-2 inhibitors in dementia prevention. Standardized methodologies are needed to ensure consistency and comparability.

Most studies focus on diabetic populations, particularly those with type 2 diabetes. While diabetes is a known risk factor for dementia, it is unclear whether SGLT-2 inhibitors offer similar neuroprotective benefits in non-diabetic individuals or those with other metabolic conditions. Expanding research to include diverse populations could clarify the generalizability of these findings.

SGLT-2 inhibitors also carry risks, including urinary tract infections, hypoglycemia (when combined with other antidiabetic agents), dehydration, and electrolyte imbalances [19]. Patients with cardiovascular disease or impaired renal function require careful monitoring, and there is an increased risk of ketoacidosis, especially in those with low insulin levels or on low-carbohydrate diets [85,86]. Clinicians should thoroughly assess patients before initiating treatment, balancing efficacy and safety based on individual risk factors.

## 6. Conclusions

Current research indicates that SGLT-2 inhibitors, such as empagliflozin and dapagliflozin, have potential neuroprotective effects that could reduce the risk of dementia and improve cognitive function in patients with type 2 diabetes. These effects are likely due to improvements in glycemic control, reduction in oxidative stress, and decreased formation of advanced glycation end-products (AGEs), all of which are associated with neurodegeneration. However, many studies are limited by small sample sizes and short follow-up periods, which may not fully capture the long-term benefits and potential risks of SGLT-2 inhibitors on cognitive health. To establish more definitive evidence, larger-scale, long-term randomized controlled trials (RCTs) are necessary. These studies should aim to include diverse populations and focus on understanding the mechanisms behind the neuroprotective effects of SGLT-2 inhibitors. By addressing these limitations, future research can provide clearer guidelines for the use of SGLT-2 inhibitors in preventing and managing dementia in diabetic patients, ultimately improving therapeutic strategies for cognitive health.
